# Research Progress on the GIP/GLP-1 Receptor Coagonist Tirzepatide, a Rising Star in Type 2 Diabetes

**DOI:** 10.1155/2023/5891532

**Published:** 2023-04-15

**Authors:** Zijun Ma, Kaiqin Jin, Mengmeng Yue, Xin Chen, Jun Chen

**Affiliations:** ^1^Sinopharm Dongfeng General Hospital, Hubei University of Medicine, Daling Road 16, Shiyan, Hubei 442000, China; ^2^Department of Cardiology, The Second Hospital of Anhui Medical University, Furong Road 678, Hefei, Anhui 230601, China

## Abstract

Type 2 diabetes mellitus (T2DM) is a chronic progressive metabolic disease that has become a growing health problem worldwide, and the dangers of hyperglycemia and its chronic complications have long been considered a goal of diabetes treatment. In recent years, tirzepatide has become the first dual GIP/GLP-1R agonist approved for the treatment of diabetes mellitus in the United States as a new hypoglycemic medicine. Its hypoglycaemic and weight loss effects have been demonstrated in several large clinical trials, and there is also evidence that it has great potential for cardiovascular protection. In addition, the very concept of synthetic peptides opens up many unknown possibilities for tirzepatide. Ongoing trials (NCT04166773) and evidence suggest that it appears to be a promising drug in the areas of NAFLD, renal, and neuroprotection. Based on preclinical studies and clinical trials, the aim of this article is to discuss the latest clinical developments in tirzepatide, to focus on its differences with other incretin therapies, and to suggest future possibilities and mechanisms of tirzepatide therapy.

## 1. Introduction

Type 2 diabetes mellitus (T2DM) is a prevalent metabolic condition characterized by insulin resistance and inadequate insulin production as a result of a decline in pancreatic beta-cell activity [1]. Chronic hyperglycemia is a risk factor for cardiovascular disease, retinopathy, chronic kidney disease, stroke, and Alzheimer's disease. As a result, it is also a chronic disease with significant morbidity and mortality rates over the world, with the number of individuals affected increasing at an alarming rate every day and anticipated to reach 700 million by 2045 [2]. Controlling hyperglycemia and preventing the chronic complications of diabetes have long been recognized as the main goals of diabetes management. For decades, incretin has been gaining attention in the treatment of diabetes because of its effective hypoglycemic and cardiometabolic effects. Tirzepatide, a glucose-dependent insulinotropic peptide (GIP) and human glucagon-like peptide-1 (GLP-1) receptor agonist, was recently approved to treat type 2 diabetes. It is the first injectable antihyperglycemic drug with dual action (GIP/GLP-1 receptor agonism). The purpose of this review is to discuss the latest clinical developments in dual GIP and GLP-1 receptor agonists (tirzepatide) based on preclinical studies in animal models and clinical trials in humans, to highlight its differences from other antidiabetic drugs, and to suggest future possibilities and mechanisms for tirzepatide therapy.

## 2. Incretin-Based Therapy

**Figure 1:**
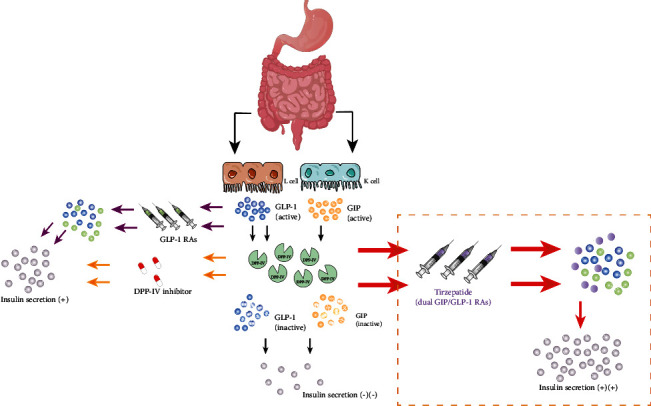
Drug mechanism of action of GLP-1 analogues, DPP-IV inhibitors, and GIP/GLP-1 receptor agonists.

Previous research has shown that in healthy subjects with normal glucose tolerance given either oral or intravenous glucose, overall plasma insulin secretion levels were significantly higher with oral glucose compared to intravenous glucose; at similar blood glucose concentrations, incretin can account for up to 70% of the total insulin secretory response. However, in T2DM patients, incretin accounts for just 20% of total insulin secretion due to decreased incretin secretion and dysfunctional receptor or postreceptor signaling, resulting in a decreased postprandial insulin and C-peptide secretion response, which contributes to hyperglycemia [3]. Incretin is known to be mostly composed of glucagon-like peptide 1 (GLP-1) and glucose-dependent insulinotropic peptide (GIP), which are released by intestinal L and K cells, respectively. GIP and GLP-1, however, can be inactivated in vivo within minutes by dipeptidyl peptidase IV (DDP-IV) [4]. Therefore, based on this therapeutic target, GLP-1 receptor analogs (GLP-1 RAs) (which increase GLP-1 concentrations but are not broken down by DPP-IV) and DPP-IV inhibitors (which inhibit DPP-IV and prolong the half-life of GLP-1) are ideal new options in antidiabetic therapy (Figure 1). Unfortunately, GIP, the first enteric insulin identified, remained underappreciated for many years due to its limited response to pancreatic cells and insulin release in T2DM patients.

## 3. Tirzepatide

**Figure 2:**
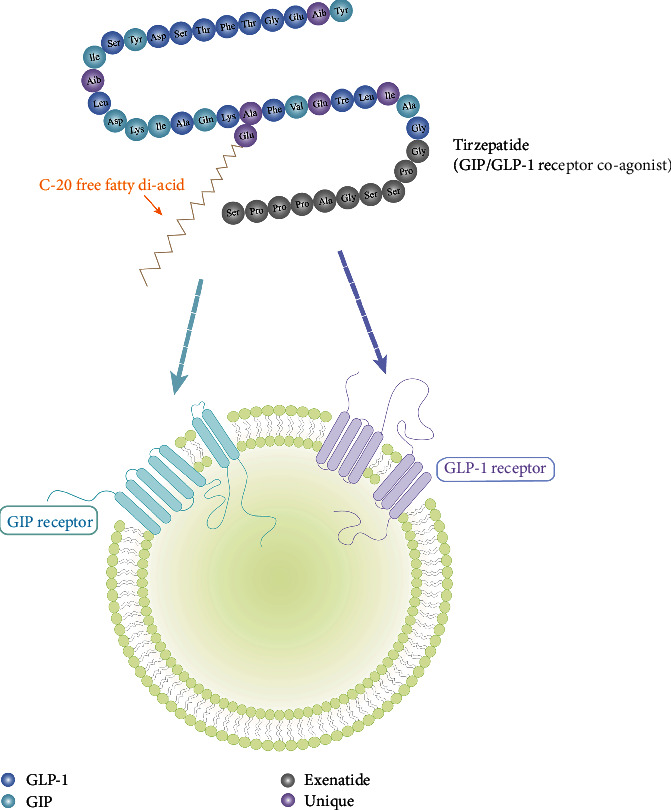
The structure of tirzepatide.

**Figure 3:**
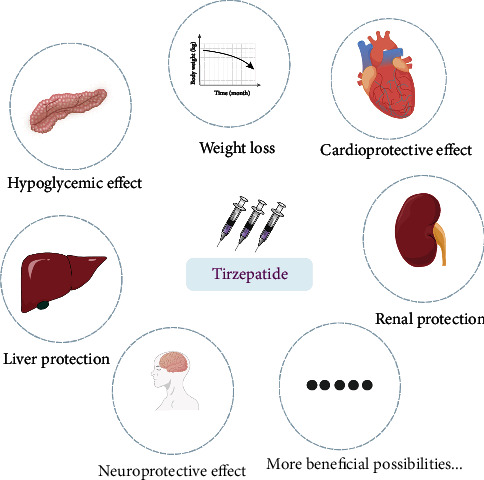
Multiple effects of tirzepatide.

It was first approved for the treatment of type 2 diabetes in the USA in May 2022 [5]. Tirzepatide is a novel single-molecule dual agonist for GLP-1 and GIP receptors; it is a GIP/GLP-1 linear chimeric peptide consisting of 39 amino acids; the structure is based on the natural GIP sequence, which is suffixed to a lysine residue via a fatty diacid molecule at the C20 position, and contains two noncoding amino acid residues (Aib, *α*-aminoisobutyric acid). In addition, the C-terminal structure of the amidated peptide chain facilitates albumin binding and a longer half-life in vivo, which makes it possible to administer the drug only once a week [6](Figure 2). In fact, tirzepatide is a biased agonist of GIPR/GLP-1R. It has a comparable affinity to GIP, but the molecule binds GLP-1R with significantly weaker affinity than natural GLP-1, an imbalance that favors GIP over GLP-1 to maximize the GIP effect in the dose range while achieving an effective and tolerable GLP-1 effect [7]. As the first approved dual agonist of the GIP and GLP-1 receptors on the market, tirzepatide is considered to be a promising drug against type 2 diabetes and obesity. Additionally, there are additional opportunities for clinical application (Figure 3).

### 3.1. Hypoglycemic Effect

The phase III (SURPASS) program is a class of trials designed to evaluate the efficacy and safety of tirzepatide in the treatment of patients with type 2 diabetes. The primary endpoint of the trial was the change in glycated hemoglobin (HbA1c) relative to baseline. Secondary endpoints included weight, fasting glucose, percentage of participants meeting different stratification criteria for HbA1c and weight, and safety events, despite the fact that several trials are currently continuing. However, when compared to the GLP-1RAs, the SURPASS-2 trial results showed that tirzepatide reduced glycated hemoglobin and fasting serum glucose levels more than semaglutide at all dosages (5, 10, and 15 mg) [8]. Participants in tirzepatide groups had statistically significant mean reductions in HbA1c from baseline compared with those in the dulaglutide group, which began at week 4, were near the maximum around week 24, and were maintained until week 52. At week 52, mean HbA1c was 5.8% (40 mmol/mol) for 5 mg, 5.6% (38 mmol/mol) for 10 mg, and 5.4% (35 mmol/mol) for 15 mg of tirzepatide compared with 6.9% (52 mmol/mol) for dulaglutide [9]. In contrast to insulin analogs, the SURPASS-3 and 4 trials compared the efficacy of tirzepatide with that of degludec and glargine insulin, respectively, and showed that tirzepatide had a greater hypoglycemic effect than insulin treatment and that a higher proportion of participants achieved an HbA1c < 7.0% at the end of the trial for all doses [10, 11]. Overall, tirzepatide outperforms GLP-1RAs and insulin in terms of blood glucose management, and there are compelling reasons to assume that tirzepatide may be the next antidiabetic medication star.

### 3.2. Weight Reduction Effect

Obesity is recognized as a risk factor for T2DM. In patients at high risk of developing antecedent diabetes and/or metabolic syndrome, weight loss is effective in preventing progression to type 2 diabetes mellitus (T2DM) and improving cardiovascular risk factors [12]. Many of the current antidiabetic drugs have both glucose-lowering and weight-lowering effects, such as dulaglutide and dapagliflozin. Strong research suggests that obesity management can delay the progression of prediabetes to type 2 diabetes and facilitate the treatment of type 2 diabetes [13–15]. In addition, the American Diabetes Association (ADA) also recommends weight loss based on intensive lifestyle interventions to achieve optimal control of traditional cardiovascular risk factors in overweight or obese adults with type 2 diabetes [16]. According to the SURPASS program's findings, tirzepatide significantly reduced body weight in type 2 diabetics when compared to baseline, and all dosages of tirzepatide caused greater weight loss when compared to GLP-1RAs [8, 10].

The SURMOUNT-1 trial in the obese population found that participants at all dosages (5 mg, 10 mg, and 15 mg) had 11.9% to 17.9% lower body weight than baseline in obese or overweight individuals without diabetes. At the same time, weight loss with tirzepatide was accompanied by a significant improvement in cardiovascular and metabolic risk factors compared to placebo (including waist circumference, systolic and diastolic blood pressure, fasting insulin levels, and blood lipid levels) [17]. Semaglutide is a GLP-1R single agonist, similar to tirzepatide, that significantly improves hyperglycemia and obesity and has been approved by the FDA in 2021 as a weight loss drug for chronic weight management [18]. Unfortunately, there are no trials evaluating the comparative weight loss and safety assessment of tirzepatide and semaglutide in obese or overweight populations.

### 3.3. Cardiovascular Protection

Smoking, being overweight or obese, diabetes, dyslipidemia, and hypertension are all recognized cardiovascular risk factors [19]. Current research suggests that tirzepatide, like GLP-1RAs, may enhance numerous cardiovascular risk factors. In a phase 2b trial evaluating the effects of tirzepatide in type 2 diabetic population, Wilson et al. observed a time-dependent dose-dependent reduction in levels of triglycerides, apoB, apoC-III, and reduced levels of triglyceride-rich lipoprotein particles (TRLP) and low-density lipoprotein particles [20]. Furthermore, in the high cardiovascular-risk population of the SURPASS-4 trial, reductions in triglycerides, LDL cholesterol, and total cholesterol, and increases in HDL cholesterol were observed in all of the tirzepatide participating groups [10]. Furthermore, despite the lack of separate efficacy analyses for hypertensive patients, all participants in the SURPASS study in the tirzepatide group had a drop in systolic blood pressure. In addition, Sttar et al. recently found in a meta-analysis of predefined cardiovascular event risks for all randomized controlled trials in the SURPASS program, compared to the control group, tirzepatide had a lower risk of MACE-4 (cardiovascular death, myocardial infarction, stroke, and unstable angina) [21]. The SURPASS-CVOT trial is currently underway to evaluate the impact of tirzepatide and dulaglutide on significant cardiovascular adverse events in type 2 diabetes patients (NCT04255433) and to assess cardiovascular safety more completely. Cardiovascular diseases include heart failure, atherosclerotic cardiovascular diseases, and other related diseases. We also look forward to whether there will be exciting results in the future.

## 4. Possibilities for the Future

### 4.1. Nonalcoholic Fatty Liver Disease

Nonalcoholic fatty liver disease (NAFLD) and T2DM have a significant reciprocal association. NAFLD raises the risk of T2DM and associated consequences, while T2DM itself is a risk factor for steatohepatitis (NASH) and liver-related mortality in people with NAFLD. Both of these multisystem diseases lead to elevated glucose levels and insulin resistance, resulting in lipid accumulation and oxidative and inflammatory changes in hepatocytes [22]. The majority of patients with T2DM are reported to have NAFLD/NASH, which may further increase their cardiometabolic risk [23]. Current guidelines advocate screening for NAFLD in all patients with T2DM.

In recent years, numerous antidiabetic drugs, such as pioglitazone and GLP-1RAs, have exhibited improvements in liver enzymes; both reduce cardiovascular risk and improve liver histology in individuals with NAFLD [24]; SGLT2 receptor inhibitors may also improve the metabolic abnormalities of NAFLD to some extent [25]. Mechanistically, the protective effect of GIP and GLP-1 on the liver is indirect, as hepatocytes do not express GIP and typically GLP-1, and the role of GIP and GLP-1 expression in the liver is controversial [26–29]. However, it is worth noting that an effective treatment for NAFLD is weight loss, which has been shown to improve hepatic steatosis and fibrosis [30]. GIP can enhance insulin secretion in the postprandial state and increase the anabolic effect of insulin in adipose tissue, thus promoting adipogenesis and preadipocyte differentiation [31, 32], while GLP-1RA can control blood glucose and lower body weight, reduce postprandial lipoprotein secretion, decrease systemic tissue inflammation, and help improve hepatic lipid metabolism disorders [33–35], which has some beneficial effects on NAFLD. Therefore, considering the direct role of GIP and GLP-1 in adipose tissue, this effect on adipose tissue may also be beneficial in NAFLD; in other words, the tirzepatide may have some indirect effect on the protection of the liver.

In a substudy of SURPASS-3 involving patients with type 2 diabetes and a liver index more than 60, tirzepatide was found to lower liver fat content considerably compared to insulin [36]; in addition, a post hoc analysis of phase 2 clinical trial by Hartman et al. showed that in the T2DM population, high doses of the dual GIP/GLP-RA agonist, tirzepatide, significantly improved K-18, Pro-C3, and lipocalin compared to placebo, while ALT and AST were dose-dependently reduced relative to baseline, but not to a greater extent than placebo [27]. Due to the limitations of post hoc analyses, although these results do not prove the effectiveness of tirzepatide in patients with NASH, they may provide support for the possible future treatment of NAFLD. Also, preclinical studies have shown that the dual combination of GIP and glucagon (GCG) agonists has a more pronounced reduction in hepatic triglycerides and improvement in liver histological disease activity scores than GLP-1RA (liraglutide). Compared with GLP-RA, the dual agonist tirzepatide had more potent hypoglycemic and weight-loss effects [37]. Although a comparison of the efficacy of GLP-RA with that of telopeptide in NAFLD is lacking, it is theorized that tirzepatide may have superior efficacy. A randomized controlled phase 2 study to explore the use of tirzepatide as a treatment for nonalcoholic steatohepatitis (NASH) may also provide strong evidence for this possibility. (NCT04166773).

### 4.2. Renal Protective Effect

The major cause of chronic kidney disease is diabetes. Hyperglycemia and diabetes-related metabolic disorders can lead to kidney damage, renal function deteriorates with time, resulting in glomerular hyperfiltration, severe proteinuria, and a further decline in the glomerular filtration rate until end-stage renal disease and dialysis [38]. Most people with diabetes will eventually develop kidney disease. In the therapy of diabetic patients, strict glycemic control and prevention or delay of renal damage might have a favorable prognostic effect.

Inflammation is an underlying condition for the development of diabetic nephropathy, and proinflammatory advanced oxidative protein products (AOPP) induce proteinuria and apoptosis of renal podocytes, further leading to glomerulosclerosis [39]. Extensive research has been conducted on GIP's impact on inflammatory processes. In an animal model, daily GIP injections decreased proinflammatory cytokines (IL-6, interleukin-1, and TNF-*α*) while increasing lipocalin levels [40], while GLP-1 receptor activation upregulates the second messenger cyclic adenosine monophosphate (cAMP) and activates protein kinase A (PKA). By inhibiting NADPH oxidase, the increased cAMP and PKA can reduce oxidative kidney damage [41]. It has also been shown that GLP-1RAs lowers the expression of several proinflammatory biomarkers in animal models of diabetic nephropathy [42]. In addition, a post hoc analysis of SURPASS-4 by Heerspink et al. shows that in participants with type 2 diabetes mellitus with high cardiovascular risk, those receiving the GIP/GLP-1R dual agonist, tirzepatide, had a significantly lower rate of composite renal endpoints, a significantly slower rate of decline in eGFR and a significantly lower urinary albumin creatinine value compared to those receiving glargine insulin [36]. Studies on the efficacy and safety of tirzepatide in patients with diabetic nephropathy are not available, but there is much indirect evidence to support the renal protective effect of tirzepatide.

### 4.3. Neurodegenerative Disease

Insulin resistance in the brain as a feature of AD was first proposed in an earlier study by Hoyer and Nitsch [43] to explain the metabolic disorders of the brain [43]. A growing body of research suggests that Alzheimer's disease (AD) is a brain-specific DM (type 3 diabetes) [44, 45]. AD shares many pathophysiological characteristics with T2DM, including insulin resistance, inflammatory stress, and amyloid-*β* accumulation [46]. Among these common features, insulin resistance is one of the key underlying mechanisms, suggesting that treatments to attenuate T2DM pathology may be successful in treating neuroinflammatory and neurodegenerative pathologies.

Incretin has been proposed as a possible new treatment option for AD due to its brain-protective properties. In humans and mice, GIPR and GLP-1R are coexpressed in the arcuate nucleus and some hypothalamic cells in the dorsomedial hypothalamus and other brain regions [47, 48]. GIP is widely present in multiple regions of the rat brain, including the hippocampus, brainstem, cerebral cortex, and cerebellum [49]. On the one hand, the effect of GIP signals on weight loss is transmitted through the central nervous system [47]; on the other hand, GIP may have a neuroprotective effect on animal models of neurodegenerative diseases such as Alzheimer's disease and Parkinson's disease by promoting neurogenesis [50]. GLP1 in the brain is involved in various biological reactions such as stress response, aversion, anorexia, hypothalamic pituitary function, neuroinflammation, and neuroprotection [51–53]. Extensive preclinical studies have shown that incretin-based drugs can achieve significant cerebral protection and reduce neuronal loss by reducing synaptic loss and amyloid plaque load, decreasing hyperphosphorylation of *τ* proteins, and anti-inflammatory effects [54]. In a preliminary study testing liraglutide in 200 AD patients, it was shown that after 12 months of liraglutide treatment, the liraglutide group had less reduction in temporal lobe volume (*p* < 0.001) and less reduction in total grey cortical volume compared to placebo. Moreover, MRI scans of the brain revealed that liraglutide prevented neuronal loss [55]. Similarly, as a dual GIP/GLP-1 receptor agonist, tirzepatide has shown some preliminary effects in preclinical studies in animal models. Zhang et al. demonstrated that DA-5CH (GIP/GLP-1 dual agonist) can cross the blood-brain barrier faster than semaglutide and may show better results in neurodegenerative diseases [56]. Moreover, in the SPP/PS1 transgenic AD mouse model, therapy with DA5-CH improved the memory function of AD animals and decreased the levels of amyloid senile plaques and phosphorylated tau protein in the hippocampus [57]. Overall, although more convincing clinical evidence is needed, incretin-based therapy with tirzepatide is considered to have great potential for neuroprotection.

## 5. Adverse Effects

Similar to GLP-1RAs, the most common adverse effects of tirzepatide are gastrointestinal discomfort, including nausea, diarrhea, and vomiting. It is worth noting that telopeptide had a lower frequency of gastrointestinal side effects than GLP-1R single agonists [10]. This phenomenon may be the result of its unbalanced dual agonistic effect, favoring GIPR over GLP-1R activity [7], because dose escalation of selective GLP-1R agonists induces undesirable gastrointestinal effects. Such effects have not been described for GIP, even in an infusion study where the concentration of GIP reached the estimated free drug levels of the higher tirzepatide doses [58], which allowed tirzepatide to achieve an effective and tolerable GLP-1 response while maximizing GIP effects. Although tirzepatide had a lower rate of gastrointestinal adverse events than GLP-1R monoagonists, the adverse effects of tirzepatide remained dose-dependent. The incidence of gastrointestinal discomfort increased when the dose was increased, but frequently during the transitional phase [8]. All glucose-lowering drugs have a risk of hypoglycemia, so those with superior glucose-lowering effects deserve more attention for their risk of hypoglycemia. Previous studies have shown that tirzepatide has a significant effect on blood glucose, which has led to concerns about whether tirzepatide increases the risk of hypoglycemia compared to other drugs. Interestingly, we discovered that the risk of hypoglycemia caused by tirzepatide was reduced when it was administered without insulinotropic drugs or insulin [10]. However, tirzepatide is contraindicated in individuals with a personal or family history of medullary thyroid cancer or in patients with multiple endocrine neoplasia type 2 syndromes, and a warning concerning the risk of thyroid C-cell tumors is noted in its prescribing information.

## 6. Prospects and Outlook

Tirzepatide was the first dual GIP/GLP-1R agonist to be approved for the treatment of diabetes in the United States as a novel glucose-lowering medication. The very concept of this synthetic peptide allows for many unknown possibilities for tirzepatide. In the SURPASS study, with a high margin over insulin and GLP-1RAs, tirzepatide was shown to significantly control blood glucose and body weight without causing hypoglycemia. The advantage of a weekly dose also greatly increases patient compliance. In the SURMOUNT-1 study, the excellent weight loss of tirzepatide in obese individuals without diabetes opens up novel possibilities for the pharmacological treatment of obesity and will provide new therapy choices for diabetes and obesity. Similarly, a number of correlative studies in cardiometabolic diseases, which are of great interest, have shown good promise for tirzepatide. We expect that the results of the future SURPASS-CVOT trial will support this suspicion and confirm the cardiovascular safety of tirzepatide. However, more clinical studies are needed to fully understand the clinical efficacy and prognostic effects of tirzepatide. For example, in terms of weight reduction, considering that tirzepatide has shown a high level of weight loss in several studies, we look forward to a comparative clinical study of tirzepatide versus semaglutide for the treatment of obesity, providing a better option for patients than semaglutide, which is already approved for the chronic management of obesity; diabetes is often associated with a variety of comorbidities, and the potential indications for tirzepatide (e.g., nonalcoholic steatohepatitis, nephroprotection, and cerebroprotection) will be an important next step to address; additionally, the glucose-lowering effect and safety of metformin as a basic drug for patients with type 2 diabetes, in combination or not, also deserve our attention; in any case, the clinical success of this dual agonist will give increasing impetus to further research on multiple agonists and may lead to even greater breakthroughs in the treatment of type 2 diabetes and its comorbidities.

## Data Availability

There is no raw data associated with this review.
